# Long-Term Functional Outcomes After Pediatric Intensive Care Unit Admission for Bronchiolitis: A 12-Month Cohort Study

**DOI:** 10.3390/children13050636

**Published:** 2026-05-02

**Authors:** Paula Sevilla Hermoso, Mireia Mor Conejo, Carme Alejandre, Laia Roig Cortes, Omar Rodriguez, Francisco José Cambra Lasaosa, Iolanda Jordan, Mònica Balaguer

**Affiliations:** 1 Pediatric Intensive Care Unit, Sant Joan de Déu Hospital, 08950 Barcelona, Spain; paula.sevilla@sjd.es (P.S.H.); mireia.mor@sjd.es (M.M.C.); lairocortes2@gmail.com (L.R.C.); omar.rodriguez@sjd.es (O.R.); franciscojose.cambra@sjd.es (F.J.C.L.); iolanda.jordan@sjd.es (I.J.); monica.balaguer@sjd.es (M.B.); 2 Institut de Recerca Sant Joan de Déu (IRSJD), University of Barcelona, 08007 Barcelona, Spain; 3 Faculty of Medicine, University of Barcelona, 08036 Barcelona, Spain; 4 Faculty of Medicine, University of Vic-Central University of Catalonia (UVic-UCC), 08500 Vic, Spain

**Keywords:** pediatrics, critical care, bronchiolitis, functional status, pediatric intensive care unit

## Abstract

**Highlights:**

**What are the main findings?**

- A clinically relevant minority of children admitted to the PICU for bronchiolitis experience long-term functional decline, mainly affecting neurological status.
- Functional decline was more frequently observed in patients with underlying conditions, need for invasive mechanical ventilation, complications, and longer PICU and hospital stays.

**What are the implications of the main findings?**

- Early identification of risk factors may help target high-risk patients for closer follow-up and the implementation of preventive strategies aimed at reducing long-term functional impairment.
- Optimizing ventilation strategies and improving the management of respiratory complications may be key to improving long-term functional outcomes.

**Abstract:**

**Introduction.** Bronchiolitis is the leading cause of Pediatric Intensive Care Unit (PICU) admission for lower respiratory tract infection in infants. Although survival has improved, concerns remain regarding potential long-term functional impairments, including alterations in psychomotor development, learning, and behavior. This study aimed to describe the epidemiological and clinical characteristics of children admitted to the PICU for bronchiolitis and to evaluate their functional outcomes at 12-month follow-up. **Methods.** A retrospective descriptive cohort study was conducted, including all patients admitted to the PICU for bronchiolitis during the 2021–2022 period. Epidemiological, clinical, microbiological, and laboratory data were collected. Functional health status was assessed using the Pediatric Overall Performance Category (POPC), Pediatric Cerebral Performance Category (PCPC), and Functional Status Scale (FSS) at PICU discharge and 12 months. Changes in functional status were categorized as improved, stable, or worsened. Exploratory unadjusted analyses were performed to describe differences between outcome groups. **Results.** A total of 164 patients were included (43.9% female), with a median age of 51 days (IQR 26.25–118.5). Respiratory syncytial virus was identified in 79.7% of cases. Invasive mechanical ventilation was required in 31.1% of patients, and 45.7% developed complications during PICU admission. Mortality was 0.6%. At 12 months, functional deterioration was observed in 14.6% of patients according to POPC, 16.5% according to PCPC, and 3.6% according to FSS. Higher proportions of functional deterioration were observed among patients with underlying medical conditions, those requiring invasive mechanical ventilation, those with complications, and those with longer PICU and hospital stays, particularly in the PCPC scale. **Conclusions.** Most children admitted to the PICU for bronchiolitis showed stable or improved functional status at 12 months. However, a subset experienced functional deterioration, more frequently observed in patients with greater clinical severity and complexity during admission. These results support the need for further studies to better characterize long-term outcomes and to identify children who may benefit from closer follow-up.

## 1. Introduction

In recent years, the management of critically ill pediatric patients has witnessed significant advancements, largely attributable to enhanced instrumentation and the allocation of more comprehensive resources within healthcare settings. These improvements have not only optimized the delivery of care but have also contributed to a notable decline in mortality rates, with certain institutions reporting reductions in the range of 3–5% [1]. Admission to the PICU offers benefits in increasing patient survival, but it also carries negative consequences. It can lead to alterations in cerebral blood flow due to hemodynamic or oxygenation-ventilation changes, resulting in acute complications and long-term sequelae [2]. These may include disruptions in psychomotor development, learning, or behavior, affecting functional health.

Understanding the characteristics of patients with worse outcomes may contribute to earlier recognition of at-risk patients, allowing for timely interventions and potentially improving long-term functional health status [3,4].

This alteration can be evaluated by analyzing the functional health status through various clinical scales. These instruments should collect objective information through validated tools exploring diverse dimensions (physical, emotional, social, etc.). In the pediatric population, the utilized functional health scales are known as the Pediatric.

Overall Performance Category and Pediatric Cerebral Performance Category (POPC/PCPC) scale [5] and Functional status scale FSS [4]. The POPC/PCPC and the FSS are typically assessed at baseline, PICU discharge, hospital discharge, and follow-up. These scales are easy to apply using clinical data, show good reliability, and are independent of age. POPC and PCPC provide global and neurological assessment, respectively, while FSS offers a more detailed evaluation across multiple functional domains, including mental status, sensory and motor function, communication, and respiratory status. Their combined use allows for a broader characterization of functional outcomes in critically ill pediatric patients. Research has explored the health-related quality of life in patients admitted to the PICU due to infections, trauma, and cardiopulmonary arrest [2,6].

Bronchiolitis is an acute lower respiratory tract infection that affects children under 24 months of age [7,8]. It is most seen between 2 and 6 months of age, and represents a considerable burden on healthcare systems. Most cases occur between November and April, peaking in January and February. The main cause is respiratory syncytial virus (RSV), although other viruses may also be involved [9,10]. While the overall prognosis is favorable, approximately 2–3% of infants with a primary RSV infection require hospitalization, and 2–6% of these require admission to the PICU [11,12].

The aim of this study is to describe the epidemiological characteristics, clinical course, and functional health outcomes of patients admitted to the PICU for bronchiolitis, as well as the distribution of functional status during follow-up.

## 2. Materials and Methods

Retrospective, descriptive, and single-center cohort study including all patients admitted to PICU with a diagnosis of bronchiolitis between September 2021 and August 2022.

This study was conducted in accordance with the Declaration of Helsinki and approved by the local Institutional Review Board and the Ethics Committee of Hospital Sant Joan de Déu. Parents or legal guardians were required to sign the informed consent document before their children were included.

The collected variables were: epidemiological (age and gender), clinical variables (PRISM III severity score, BROSJOD severity score, etiology of infection, gestational history, inotropic use, invasive mechanical ventilation (IMV) use, duration of IMV, PICU length of stay, hospital length of stay), microbiological data (etiological agent and coinfections), laboratory data (hemoglobin, leukocytes, pH, bicarbonate and arterial CO2 levels, lactate levels, C-reactive Protein (CRP), procalcitonin), outcomes during admission (complications, antibiotic use, need for organ support) and an assessment of functional status using FSS, POPC, and PCPC at admission and at 12 months after discharge.

Data were analyzed using SPSS® version 26.0. Statistical analysis was primarily descriptive. Qualitative variables were expressed as absolute frequencies and percentages and quantitative variables as means and standard deviations or medians and interquartile ranges (IQR), depending on their distribution assessed using the Kolmogorov–Smirnov test. Changes in functional status between PICU discharge and 12-month follow-up were described as proportions of patients who improved, worsened, or remained stable according to each scale.

In addition, exploratory bivariate analyses were performed to describe differences in clinical and epidemiological variables according to functional outcome groups (worsened vs. improved/equal) for each functional scale (POPC, PCPC, and FSS). Qualitative variables were compared using the chi-square test or Fisher’s exact test, as appropriate, and continuous variables were compared using Student’s t-test or Mann–Whitney U test, depending on data distribution. The significance level was set at 0.05. Given the limited number of outcome events, particularly for functional deterioration in some scales (e.g., FSS), multivariable modeling was not considered reliable due to the risk of model overfitting and unstable estimates. Therefore, the analyses were restricted to descriptive and unadjusted comparisons.

## 3. Results

A total of 164 patients were included, of whom 72 (43.9%) were female, and 92 (56.1%) were male. The median age at admission was 51 days (IQR: 26.25–118.5). The median length of stay in the pediatric intensive care unit (PICU) was 5 days (IQR: 4–9), and the median total hospital stay was 10 days (IQR: 7–16).

The median PRISM mortality score was 0 (IQR: 0–3), and the median BROSJOD severity score was 10 (IQR: 8–11).

Prematurity was present in 29 patients (17.7%). Twenty patients (12.2%) had underlying medical conditions, including congenital heart disease (4.3%), chronic lung disease (4.3%), neurological disorders (4.3%), and other conditions (6.1%).

In terms of infectious etiology, respiratory syncytial virus (RSV) was the most frequently identified pathogen in 131 patients (79.9%), followed by rhinovirus (7.3%), metapneumovirus (5.5%), influenza (3.7%), and coronavirus (0.6%). Viral coinfection was detected in 51 patients (31.1%). No significant differences in functional outcomes were observed between patients with RSV infection and those with other viral etiologies.

During PICU admission, 75 patients (45.7%) developed complications, including respiratory distress syndrome, bacterial superinfection, atelectasis, and other respiratory and systemic complications. No individual complication showed a consistent association across functional outcome scales.

Antibiotics were administered to 100 cases (61%). Thirteen patients (7.9%) required inotropic support. The median VIS score was 0 (IQR 0–3), reflecting that most patients did not require vasoactive support, although a small proportion required inotropic therapy. Non-invasive ventilation (NIV) was used in 156 patients (95.1%), and 51 patients (31.1%) required IMV. No patients required renal replacement therapy (RRT) or extracorporeal membrane oxygenation (ECMO). One patient (0.6%) died.

Epidemiological, analytical, microbiological, and clinical data collection is shown in Table 1.

Functional status at PICU discharge and at 12 months was assessed using FSS, POPC and PCPC scales. Across all three scales, patients were categorized as improved, worsened, or unchanged.

**Figure 1:**
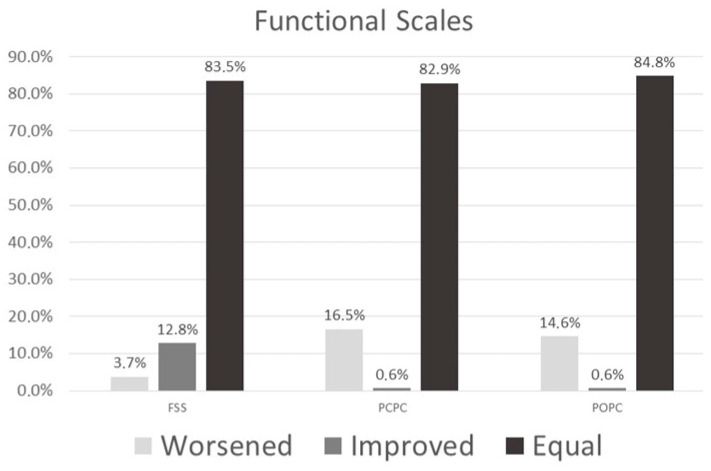
Patient’s evolution in functional health from PICU discharge to one year after discharge.

Figure 1 shows the distributions of these categories at both time points. Overall, most patients remained stable or improved over time, while a smaller proportion experienced functional deterioration.

The exploratory bivariate analysis of each variable with one of the functional health scales is shown in Table 2.

For the PCPC scale, higher proportions of baseline disease (25.9% vs. 9.5%, *p* = 0.026), need for invasive mechanical ventilation (51.9% vs. 27%, *p* = 0.011), complications (66.7% vs. 41.6%, *p* = 0.017), and longer PICU (*p* = 0.021) and hospital length of stay (*p* = 0.006) were observed among patients with functional deterioration. For the POPC scale, a lower proportion of antibiotic use was observed among patients with deterioration compared to those with stable or improved outcomes (41.7% vs. 64.3%, *p* = 0.036). No statistically significant differences were observed in the FSS scale. All findings are presented as unadjusted comparisons and should be interpreted as descriptive differences between groups rather than causal associations.

Additional exploratory analyses of individual complications showed heterogeneous patterns across the different scales. Some complications, such as respiratory distress syndrome, appeared more frequently among patients with worse functional outcomes in certain scales; however, these findings were not consistent across all analyses. Given the exploratory nature of these analyses and the limited number of events, the results are not presented in detail.

## 4. Discussion

This study describes the evolution of functional health status in children admitted to the PICU for bronchiolitis. Functional deterioration at 12 months was observed in 14.6% of patients according to the POPC scale, 16.4% according to PCPC, and 3.6% according to FSS. These proportions are consistent with previously published data. The RESTORE study [13], where the evolution of the functional status of severe bronchiolitis is assessed according to the level of sedation and other risk factors, reported functional decline in approximately 12% of patients at 6 months. In contrast, another study [14] using different assessment tools (Bayley III, ASQ, PEDI-CT, Amiel-Tison) has described higher rates of impairment, likely reflecting differences in outcome definitions and measurement instruments rather than true clinical discrepancies.

The lower proportion of functional deterioration identified by the FSS compared to the POPC and PCPC scales may be explained by differences in scale structure and responsiveness. POPC and PCPC are global categorical measures that can capture relatively small but clinically relevant shifts in functional status, whereas the FSS assesses multiple domains and requires a greater accumulation of deficits to reflect overall deterioration. These differences likely reflect inherent measurement properties rather than true differences in patient outcomes.

Higher proportions of functional deterioration were observed among patients with underlying medical conditions, those requiring invasive mechanical ventilation, those who developed complications during the PICU stay, and those with longer PICU and hospital stays. Similar findings have been reported in previous studies, where baseline comorbidity and greater illness severity were associated with worse long-term outcomes [13,14,15]. These descriptive associations likely reflect differences in disease severity rather than causal relationships.

Regarding respiratory support, invasive mechanical ventilation was more frequently observed among patients with worse functional outcomes. This finding is consistent with previous studies by Shein et al. [16] and Fischer et al. [13], which identified invasive mechanical ventilation as a marker of greater clinical severity in children with severe bronchiolitis.

Similarly, complications during PICU admission and longer lengths of stay were more commonly observed among patients with worse functional evolution. These findings are in line with previous studies of Saju et al. [15] and Leif Bjarte Rolfsjord et al. [17], suggesting that more complex clinical courses are associated with increased morbidity during follow-up. Prolonged PICU stays may therefore reflect both greater initial severity and a higher burden of complications during admission.

Regarding respiratory sequelae, a proportion of patients (7.9%) developed asthma during follow-up. This finding is consistent with previous literature, including the study by van Dijk et al. [18], which reported persistent respiratory symptoms after severe bronchiolitis requiring intensive care.

Differences in antibiotic exposure were observed across functional outcome groups in the POPC scale. However, given the descriptive design of this study, as well as the limited sample size, these findings should be interpreted with caution. Further studies are needed to clarify this association

Exploratory analyses of individual complications showed heterogeneous patterns across functional scales, without consistent associations between specific complications and functional outcomes. Given the limited number of outcome events, particularly for some scales, multivariable modeling was not considered reliable, and results should be interpreted as exploratory.

This study has several limitations. First, its single-center design may limit the generalizability of the findings. Although the sample size is relatively large for a pediatric cohort, the low number of patients with functional deterioration limits the ability to identify consistent patterns. The cohort also included patients with heterogeneous clinical characteristics and different viral etiologies, which may further affect generalizability. In addition, the lack of standardization in functional outcome measures complicates comparisons with other studies. Finally, the absence of relevant confounders such as sedation and analgesia exposure and socioeconomic variables may influence interpretations of results. Overall, these findings should be interpreted as descriptive rather than causal.

Despite these limitations, this study provides relevant data on long-term functional outcomes after severe bronchiolitis in a contemporary PICU cohort. The results suggest that although most children recover without significant impairment, a subgroup of patients is at risk of functional deterioration, particularly those with more severe clinical courses during admission.

## 5. Conclusions

In this cohort, functional deterioration at 12 months was observed in a minority of patients admitted to the PICU for bronchiolitis. Patients with worse functional outcomes more frequently had features of greater clinical complexity, including underlying medical conditions, need for invasive support, complications during admission, and longer hospital stays. These findings provide a descriptive overview of functional outcomes after severe bronchiolitis and support the need for further multicenter studies with larger sample sizes to better characterize these patterns.

## Figures and Tables

**Table 1:** Descriptive of the study population.

		Variables		Values
Sex		Male, *n* (%)		92 (56.1%)
		Female, *n* (%)		72 (43.9%)
		Age in days at admission, median (IQR)		51 (26.25–118.5)
Length of stay (days)		PICU, median (IQR)		5 (4–9)
		Hospital, median (IQR)		10 (7–16)
Severity scores		PRISM III, median (IQR)		0 (0–3)
		BROSJOD, median (IQR)		10 (8–11)
RF	Prenatal	Prematurity, *n* (%)		29 (17.7%)
	Postnatal	Underlying disease, *n* (%)		20 (12.2%)
		Type of underlying disease	Cardiopathy, *n* (%)	7 (4.3%)
			Pneumopathy, *n* (%)	7 (4.3%)
			Neuropathy, *n* (%)	7 (4.3%)
			Other diseases, *n* (%)	10 (6.1%)
Etiology		RSV, *n* (%)		131 (79.9%)
		Rhinovirus, *n* (%)		12 (7.3%)
		Metapneumovirus, *n* (%)		9 (5.5%)
		Influenza, *n* (%)		6 (3.7%)
		Coronavirus, *n* (%)		1 (0.6%)
		Negative results, *n* (%)		3 (1.8%)
		Coinfection, *n* (%)		51 (31.1%)
		Complications during PICU stay, *n* (%)		75 (45.7%)
Treatments		Antibiotics, *n* (%)		100 (61%)
		Ionotropic, *n* (%)		13 (7.9%)
		Median VIS, median (IQR)		0 (0–3)
		NIV, *n* (%)		156 (95.1%)
		IMV, *n* (%)		51 (31.1%)
		RRT, *n* (%)		0 (0%)
		ECMO, *n* (%)		0 (0%)
Mortality. *n* (%)				1 (0.6%)

PICU: pediatric critical care unit, PRISM: pediatric risk score mortality, BROSJOD: bronchiolitis score Sant Joan de Déu, RF: Risk factors, RSV: respiratory syncytial viruses, NIV: non-invasive ventilation, IMV: invasive mechanical ventilation, VIS: Vasoactive-Ionotropic Score, RRT: renal replacement treatment, ECMO: Extracorporeal membrane oxygenation.

**Table 2:** Presence of variables according to the evolution of the FSS scale, PCPC scale, and POPC scale, from PICU discharge to one year after admission.

**FSS**	**FSS Worsened** **(*n* = 6)**	**FSS Improved/Equal** **(*n* = 158)**	***p*** **-Value**
Baseline disease	1 (16.7%)	19 (12%)	0.548
Need for IMV	2 (33.3%)	49 (31%)	1.000
Need for NIV	6 (100%)	150 (94.9%)	1.000
Complications	3 (50%)	72 (45.6%)	1.000
PICU days	6.5 (4–11.25)	5 (3–9)	0.395
Hospital days	12 (9.5–16)	10 (7–16)	0.332
Antibiotics	3 (50%)	97 (61.4%)	0.679
Ionotropic	0	13 (8.2%)	1.000
VIS score	0 (0–0)	0 (0–0)	0.466
**PCPC**	**PCPC Worsened** **(*n* = 27)**	**PCPC Improved/Equal** **(*n* = 137)**	***p*** **-Value**
Baseline disease	7 (25.9%)	13 (9.5%)	0.026 *
Need for IMV	14 (51.9%)	37 (27%)	0.011 *
Need for NIV	26 (96.3%)	130 (94.9%)	1.000
Complications	18 (66.7%)	57 (41.6%)	0.017 *
PICU days	9 (4–11)	5 (3–8)	0.021 *
Hospital days	14 (9–19)	10 (7–13.5)	0.006 *
Antibiotics	18 (66.7%)	82 (59.9%)	0.679
Ionotropic	4 (14.8%)	9 (6.6%)	0.232
VIS score	0 (0–0)	0 (0–0)	0.163
**POPC**	**POPC Worsened** **(*n* = 24)**	**POPC Improved/Equal** **(*n* = 140)**	***p*** **-Value**
Baseline disease	4 (16.7%)	16 (11.4%)	0.500
Need for IMV	5 (20.8%)	46 (32.9%)	0.240
Need for NIV	24 (100%)	132 (94.3%)	0.605
Complications	9 (37.5%)	66 (47.1%)	0.381
PICU days	5 (3.12–8.5)	6 (4–9)	0.519
Hospital days	11 (7–13.75)	10 (7–16.75)	0.776
Antibiotics	10 (41.7%)	90 (64.3%)	0.036 *
Ionotropic	2 (8.3%)	11 (7.9%)	1.000
VIS score	0 (0–0)	0 (0–0)	0.992

IMV: invasive mechanical ventilation, NIV: non-invasive ventilation, PICU: pediatric critical care unit, VIS: Vasoactive-Ionotropic Score. * *p*-value < 0.05.

## Data Availability

The data presented in this study are available on request from the corresponding author.
